# Hepatitis D infection induces IFN-β-mediated NK cell activation and TRAIL-dependent cytotoxicity

**DOI:** 10.3389/fimmu.2023.1287367

**Published:** 2023-12-08

**Authors:** Christopher Groth, Jovana Maric, Irene Garcés Lázaro, Tomáš Hofman, Zhenfeng Zhang, Yi Ni, Franziska Keller, Isabelle Seufert, Maike Hofmann, Christoph Neumann-Haefelin, Carsten Sticht, Karsten Rippe, Stephan Urban, Adelheid Cerwenka

**Affiliations:** ^1^ Department of Immunobiochemistry, Mannheim Institute for Innate Immunoscience (MI3), Medical Faculty Mannheim, Heidelberg University, Mannheim, Germany; ^2^ Department of Infectious Diseases, Molecular Virology, Heidelberg University, Heidelberg, Germany; ^3^ German Center for Infection Research Deutsches Zentrum für Infektionsforschung (DZFI) - Heidelberg Partner Site, Heidelberg, Germany; ^4^ Department of Medicine II (Gastroenterology, Hepatology, Endocrinology and Infectious Diseases), Faculty of Medicine, Medical Center-University of Freiburg, Freiburg, Germany; ^5^ Division of Chromatin Networks, German Cancer Research Center Deutsches Krebsforschungszentrum (DKFZ) and Bioquant, Heidelberg, Germany; ^6^ Medical Research Center, Medical Faculty Mannheim, University of Heidelberg, Mannheim, Germany; ^7^ European Center for Angioscience (ECAS), Medical Faculty Mannheim, Heidelberg University, Mannheim, Germany

**Keywords:** NK cells, HDV, TRAIL, HBV, IFN-beta

## Abstract

**Background and aims:**

The co-infection of hepatitis B (HBV) patients with the hepatitis D virus (HDV) causes the most severe form of viral hepatitis and thus drastically worsens the course of the disease. Therapy options for HBV/HDV patients are still limited. Here, we investigated the potential of natural killer (NK) cells that are crucial drivers of the innate immune response against viruses to target HDV-infected hepatocytes.

**Methods:**

We established *in vitro* co-culture models using HDV-infected hepatoma cell lines and human peripheral blood NK cells. We determined NK cell activation by flow cytometry, transcriptome analysis, bead-based cytokine immunoassays, and NK cell-mediated effects on T cells by flow cytometry. We validated the mechanisms using CRISPR/Cas9-mediated gene deletions. Moreover, we assessed the frequencies and phenotype of NK cells in peripheral blood of HBV and HDV superinfected patients.

**Results:**

Upon co-culture with HDV-infected hepatic cell lines, NK cells upregulated activation markers, interferon-stimulated genes (ISGs) including the death receptor ligand tumor necrosis factor-related apoptosis-inducing ligand (TRAIL), produced interferon (IFN)-γ and eliminated HDV-infected cells via the TRAIL-TRAIL-R2 axis. We identified IFN-β released by HDV-infected cells as an important enhancer of NK cell activity. In line with our *in vitro* data, we observed activation of peripheral blood NK cells from HBV/HDV co-infected, but not HBV mono-infected patients.

**Conclusion:**

Our data demonstrate NK cell activation in HDV infection and their potential to eliminate HDV-infected hepatoma cells via the TRAIL/TRAIL-R2 axis which implies a high relevance of NK cells for the design of novel anti-viral therapies.

## 1 Introduction

The HDV affects at least 12 million people worldwide (1). The HDV is a small, single-stranded negative-sense RNA virus, capable of infecting hepatocytes. For the formation and secretion of progeny virus the HDV requires the envelope protein of the HBV. Recent studies demonstrated cell division-mediated intrahepatic spread of HDV-mono-infected cells in humanized mouse models and *in vitro* (2–4). It was reported recently that similar to HBV, HDV uses the human sodium taurocholate co-transporting polypeptide (hNTCP) receptor to enter hepatocytes (5). Thus, ectopic expression of the NTCP in hepatic cell lines opened up the possibility to study HDV infection *in vitro*, and to gain insights into their crosstalk with other cells in co-culture. In 2020, Bulevirtide/Hepcludex (formerly called Myrcludex B), a peptidic inhibitor that specifically targets the NTCP, was conditionally approved in Europe, offering a valuable treatment option for HDV patients (6). Understanding the immune response is required to extend the repertoire of current therapy options (7). NK cells are abundant in liver tissue and known for their anti-viral activity (8). In hepatitis C virus (HCV)-infected patients, NK cells express a high amount of TRAIL and the degranulation marker CD107a, indicative of a cytotoxic state, compared to healthy donors (HDs) (9). A higher frequency of NK cells prior to therapy with IFN-α, the currently only off-label treatment for HDV patients, was positively associated with a therapy-induced reduction in HDV RNA in serum, highlighting the significance of NK cell activity in HDV infections (10). NK cells can stimulate the cellular immune responses through release of IFN-γ, or can directly eliminate virus-infected cells (11). Killing of infected cells can be mediated either through the release of cytotoxic granules containing perforin and granzyme B, or by the engagement of death receptors on target cells, TRAIL-R1/2 and Fas (12). TRAIL-mediated cytotoxicity has not only been shown to be important for the elimination of HBV-infected cells, but can also to fulfill an immunoregulatory function through the elimination of hepatitis virus-specific T cells (13). The mechanistic underpinnings of NK cell reactivity in HDV infection, however, are poorly understood. Therefore, we established co-culture models of human NK cells and three different hepatocyte cell lines susceptible to HDV infection (HepG2-hNTCP, Huh7-hNTCP, HepaRG-hNTCP), to dissect the activation, cytotoxicity and immunoregulatory role of NK cells, and validated our results in a cohort of hepatitis patients.

## 2 Materials and methods

### 2.1 HepG2-hNTCP, Huh7-hNTCP and HepaRG-hNTCP cells

Human NTCP (hNTCP) overexpressing HepG2, Huh-7 and HepaRG cells were generated as described (5, 14). HepG2-hNTCP and Huh7-hNTCP cells were kept in DMEM (Thermo Fisher Scientific, Cat.: 41965062) supplemented with 10% fetal bovine serum (FBS) (Sigma Aldrich, Cat.: S0615), 1% penicillin/streptomycin (P/S) (Life Technologies, Cat.: 15140122) and 2mM Glutamine (Life Technologies, Cat.: 25030024). HepaRG-hNTCP cells were kept in William E medium (Life Technologies, Cat.: 22551089) supplemented with 10% FBS (GE Healthcare, Cat.: SH30066.03), 2 mM L-Glutamine, 1% P/S, 5μg/ml insulin (Sigma Aldrich, Cat.: 91077C-1G) and 50 μM hydrocortisone (Sigma Aldrich, Cat.: H4881-1G).

### 2.2 Isolation of NK and T cells from buffy coats of HDs

PBMCs were enriched by a Bicoll density centrifugation (Biochrom, Cat: L 6115). After removal of thrombocytes through additional centrifugations steps, NK cells were isolated by negative selection using the Human NK cell Isolation Kit (Miltenyi Biotec, Cat.: 130-092-657) according to the manufacturer’s protocols. Purity of isolated CD3^-^CD56^+^ NK cells was > 90% among live cells as determined by flow cytometry. NK cell-depleted flow through was used for positive selection of autologous CD3^+^ T cells using the CD3 MicroBeads kit, human (Miltenyi Biotec, Cat.: 130-050-101). Purity of CD3^+^ T cells was >95% as determined by flow cytometry.

### 2.3 Generation of IFNAR KO NK cells

NK cells were isolated as described above and were kept in NK cell medium (Serum-free Stem Cell Growth Medium (CellGenix, Cat.: 20802-0500), 10% Human serum, 1% P/S, 400 U/ml IL-2) for 24 h. Cells were then transfected by electroporation (Neon^®^ electroporation system, Thermo Fisher) with the CRISPR/Cas9 ribonucleoprotein system, using *Streptococcus pyogenes* Cas9 protein with two nuclear localization signals (2NLS) (Synthego). sgRNA complex of crisprRNAs (crRNAs) targeting the IFNAR α-subunit (GCGGCTGCGGACAACACCCATGG) and the IFNAR β-subunit (GCCTATGTCACCGTCCTAGAAGG), or control crRNA, and ATTO-550-coupled 67mer tracrRNA (trans-activating crispr RNA) (IDT, Cat.: 1075928) were formed. Electroporation conditions were as previously published (15). After transfection, NK cells were kept in NK cell medium for 24 h, followed by sorting for Atto-550-expressing cells. Sorted NK cells were kept for 24 h in NK cell medium with 100 U/ml IL-2 before experiments.

### 2.4 Co-culture of NK cells with HDV-infected hepatoma cells

HepG2-hNTCP, Huh7-hNTCP or HepaRG-hNTCP cells were seeded at a density of 10^6^ cells/ml in DMEM (10% FBS, 1% P/S, 2mM Glutamine). After 24 h, medium was removed and cells were cultured in transfection medium (DMEM (10% FBS, 1% P/S, 2mM Glutamine, 2.5% dimethyl sulfoxide (DMSO), 4% polyethylene glycol (PEG)) with or without HDV. HDV was obtained by co-transfection of Huh7 cells using the pJC126 (containing the HDV antigenome) and pT7HB2.7 (containing the HBV envelope proteins) plasmids followed by purification using heparin affinity chromatography (16). 24h after infection medium was removed, cells were washed with phosphate-buffered saline (PBS) and further cultured in DMEM (10% FBS, 1% P/S, 2mM Glutamine, 2.5% DMSO). After 4 days, medium was removed and hepatoma cells were further cultured alone or in presence of NK cells or IFNAR knock out (KO) NK cells at an initial effector: target (E:T) ratio of 4:1 in DMEM (10% FBS, 1% P/S, 2mM Glutamine, 50 U/ml Interleukin (IL)-2). NK cells and hepatoma cells were analyzed at the indicated timepoints of co-culture. In addition, SPs were collected, sterile filtered (Sigma Aldrich, Cat.: SLGP033R) and stored at -80°C. For *in vitro* stimulation experiments, SPs (supernatants) were diluted 1:1 with DMEM (10% FBS, 1% P/S, 2mM Glutamine, 50 U/ml IL-2).

### 2.5 Co-culture of NK cells with HepG2-hNTCP spheroids

For spheroid generation, HepG2-hNTCP cells were seeded at a density of 10^6^ cells/ml in DMEM (10% FBS, 1% P/S, 2mM Glutamine). After 24h, medium was removed and cells were cultured in transfection medium [DMEM (10% FBS, 1% P/S, 2mM Glutamine, 2.5% DMSO, 4% PEG)] with or without HDV virus. After 24 h, SP was removed, cells were washed 1x with PBS and trypsinated. Trypsinated cells were taken up in basement membrane extract (BME) (R&D Systems, Cat.: 3433-001-R1) (10^6^ cells/ml), and seeded in a 24-well plate (500µl/well). 1ml Hepatocyte High Performance Medium (Upcyte technologies GmbH) was added on top of the BME layer, and cells were cultured for 96 h. After 96h, medium was removed and NK cells were added at an initial ratio of 4:1 in DMEM (10% FBS, 1% P/S, 2mM Glutamine).

### 2.6 Generation of the HepG2-hNTCP TRAIL-R2 KO (hNTCP-TR2 KO) cell lines

The HepG2-hNTCP cell line was lentivirally transduced with a plasmid encoding the Cas9 protein and the blasticidin-resistance gene (Addgene, Cat.: 52962). To select Cas9-expressing cells, blasticidin (5 µg/ml, Gibco, Cat.: 10166793) was added to the cell culture medium for 7 days following transduction. Gene single-guide (sg)RNA (5’ CCCATCTTGAACATACCAGG 3’) targeting TNFRSF10B (TRAIL-R2) was designed using the https://design.synthego.com/#/ website, and cloned into the HDCRISPRv1 sgRNA-expression vector, as previously described. The HepG2-NTCP-Cas9-blast cell line (resistant against blasticidin) was transfected using jetOPTIMUS (Polyplus Cat.: 117-15) (17). HDCRISPRv1 also encodes for a puromycin N-acetyltransferase, allowing subsequent selection of successfully transfected cells. After 24h, cells were treated with 2.5 µg/ml puromycin (Gibco, Cat.: A1113802) for 2 days. Afterwards TRAIL R2 negative cells were sorted using a BD FACSAria™ Fusion.

### 2.7 Flow cytometric analysis

Prior to antibody incubation, FcR block was performed on all samples according to the manufacturer’s instructions (FcR Blocking Reagent, human, Miltenyi, Cat.: 130-059-901). Live cells were identified using the Zombie NIR™ Fixable Viability Kit (Biolegend, Cat.: 423105) Zombie Aqua™ Fixable Viability Kit (Biolegend, Cat.: 423101), fixable viability dye e780 (eBioscience, Cat.: 65-0865) or the BD Horizon™ Fixable Viability Stain 700 (BD, Cat.: 564997) according to the manufacturer’s instructions. Samples were acquired on a BD LSRFortessa™ X-20 or BD FACSCelesta™. Analysis of samples was done with FlowJo v. 10.8.1. Gating for flow cytometry analysis was based on isotype stained or unstained controls. Commercial antibodies, isotype controls, recombinant human chimera proteins and secondary antibodies are listed in the **Supplementary Tables 1–4**. Data is displayed as the expression frequency or as the geometric mean fluorescence intensity (gMFI) of positive cells.

#### 2.7.1 Flow cytometric staining for NTCP

To determine NTCP expression, cells were incubated with Atto-488 labeled Bulevirtide/Hepcludex (200 nM) for 30 min at 4°C.

#### 2.7.2 Flow cytometric staining for the HDV antigen

Hepatoma cells were fixed with 4% Paraformaldehyde (PFA) for 10 min at room temperature then permeabilized with 0.5% Triton X-100 for 10 min at room temperature and afterwards stained with Atto-488- or Atto-565-coupled antibody against the hepatitis D antigen (HDAg) (Clone: FD3A7, 1:100) (18).

### 2.10 Immunofluorescence staining for the HDV antigen

Hepatoma cells were fixed with 4% PFA for 10 min at room temperature and then permeabilized with 0.5% Triton X-100 for 10 min at room temperature. Afterwards, mouse anti-HDAg monoclonal antibody, FD3A7, was used as the primary antibody and goat anti-mouse Alexa Fluor 488 (Invitrogen, Cat.: A-11001) was used as the secondary antibody (18). Nuclei were counterstained with Hoechst 33342. Images were taken with Leica DM IRB microscope (10-fold magnification). The frequency of HDAg^+^ cells was determined using the particle analysis function of ImageJ.

### 2.11 Analysis of NK cell-mediated cytotoxicity towards HDV-infected cells

Frequency of dead HepG2-hNTCP cells with impaired membrane integrity was determined using the Zombie NIR™ Fixable Viability Kit (Biolegend, Cat.: 423105) or the BD Horizon™ Fixable Viability Stain 700 (BD, Cat.: 564997) according to the manufacturer’s instructions. Apoptotic Huh-7 cells were determined by surface staining for Annexin V. In this case, cells were stained in Annexin binding buffer (BD, Cat.: 556454) according to the manufacturer’s instructions with the indicated antibodies for 30 min at 4°C. Measurements were performed immediately after staining. To analyze NK cell-mediated cell death of HDV-infected HepG2-hNTCP-TR2 KO cells or HepG2-hNTCP-Cas9 control cells, lactate dehydrogenase (LDH) activity was analyzed in supernatants derived from co-culture of these cells with NK cells using the LDH-Glo™ Cytotoxicity Assay kit (Promega, Cat.: J2380) according to the manufacturer’s instructions.

### 2.12 Analysis of cytokine concentration in cell culture supernatants

Amounts of IFN-α2;β;γ;λ−1/2/3 in cell culture SPs were determined using the LEGENDplex™ Human Type 1/2/3 Interferon Panel (Biolegend, Cat.: 740396) according to the manufacturer’s instructions.

### 2.13 Analysis of T cell proliferation in co-culture with NK cells

96-well flat-bottomed plates were coated with anti-CD3 (0.1µg/ml, Fisher Scientific, Cat.: 15276737) and anti-CD28 antibody (0.2 µg/ml, Invitrogen, Cat.: 16-0289-81). NK cells from non-infected or HDV-infected co-cultures were isolated using CD56 MicroBeads (Miltenyi, Cat.: 130-097-042) and co-cultured with autologous CD3^+^ T cells in Ab-coated 96-well plates at a NK:T cell ratio of 1:4 for 72h in DMEM (10% FBS, 1% P/S, 2mM Glutamine). Prior to co-culture, T cells were incubated with carboxyfluorescein succinimidyl ester (CFSE) (2nM, Invitrogen, Cat.: C34554) to asses T cell proliferation by flow cytometry.

### 2.14 Treatment of NK cells with IFNs

Isolated NK cells (1x10^6^/ml) were cultured in DMEM (10% FBS, 1% P/S, 2mM Glutamine) with or without IFN-α2 (R&D Systems, Cat.: 11100-1), IFN-β (PeproTech, Cat.: 300-02BC), IFN-λ1 (R&D Systems, Cat.: 1598-IL), IFN-λ2 (Sigma Aldrich, Cat.: SRP3060) or IFN-λ3 (R&D Systems, Cat.: 5259-IL) (2 ng/ml) for 24 h.

### 2.15 Transcriptome analysis

NK cells were collected after 48 h of co-culture with HDV-infected or non-infected HepG2-hNTCP cells. To remove residual HepG2-hNTCP cells, NK cells were isolated through positive selection with magnetic beads according to the manufacturer’s instructions (purity > 85% as determined by flow cytometry). RNA was isolated from NK cells using the RNeasy Mini Kit (Quiagen, Cat.: 74104) according to the manufacturer’s instructions. RNA libraries were prepared with the TruSeq Stranded Total RNA Gold with Ribo-Zero Plus protocol (Illumina, San Diego, USA). The libraries were sequenced 50 bp paired-end on a NovaSeq 6000 platform (Illumina, San Diego, USA). FASTQ reads were aligned using the STAR aligner (version 2.5.3a) (19). Reads were aligned to a STAR index generated from the 1000 genomes assembly, gencode 19 gene models and for a sjbdOverhang of 200. Duplicate marking was done with Sambamba (version 0.6.5) (20). Quality control analysis was performed using the samtools flagstat command (version 1.6). Read counting over exon features from gencode 19 gene models was performed with FeatureCounts (21). Both reads of a paired fragment (quality threshold of 255) were used for strand unspecific counting. The count data were transformed to TPM and FPKM expression values. For total library abundance calculations, all genes on chromosomes X, Y, MT and rRNA as well as tRNA genes were omitted. Analysis of expression data was performed with R and bioconductor using the NGS analysis package systempipeR (22). Count data was transformed using the voom function to perform linear modelling (23). Differential expression analysis was performed using the limma package in R. A false positive rate of α= 0.05 with FDR correction was taken as the level of significance. Heatmaps were created using ggplot2 package (version 2.2.1) and the complexHeatmap (version 2.0.0) (24). The pathway analysis was made with the fgsea package (25) and the enrichment browser package (26) in R using the pathway information from KEGG database (https://www.genome.jp/kegg/pathway.html).

### 2.16 Statistics

For testing of statistical significance paired or unpaired student t-test were applied using GraphPad Prism v8.0.1. In case of normalized data, significance was tested on log-transformed values. A p value of p<0.05 was considered significant. Data is shown as the mean +/- standard error of the mean (SEM).

### 2.17 Study approval statement

For the isolation of primary NK and T cells buffy coats were provided by the DRK-Blutspendedienst (according to the principles of the Declaration of Helsinki). Prior to blood donation, written consent was obtained by all donors (approved by the Ethik Kommission II of the Medical Faculty Mannheim, 87/04). Frozen PBMC of HBV, HDV/HBV patients and HDs for flow cytometric analysis of NK cells and analysis of cytokines were provided by the Freeze Biobank, University Hospital Freiburg (project number: 383/19, project number: 512/19 (project manager: Dr. Christoph Neumann-Haefelin), project number: 515/19 (project manager: Dr. Maike Hofmann) (**Supplementary Tables 5–9**). Prior to sample acquisition, all individuals provided written informed consent.

## 3 Results

### 3.1 HDV-infected hepatic cell lines trigger NK cell activation

**Figure 1:**
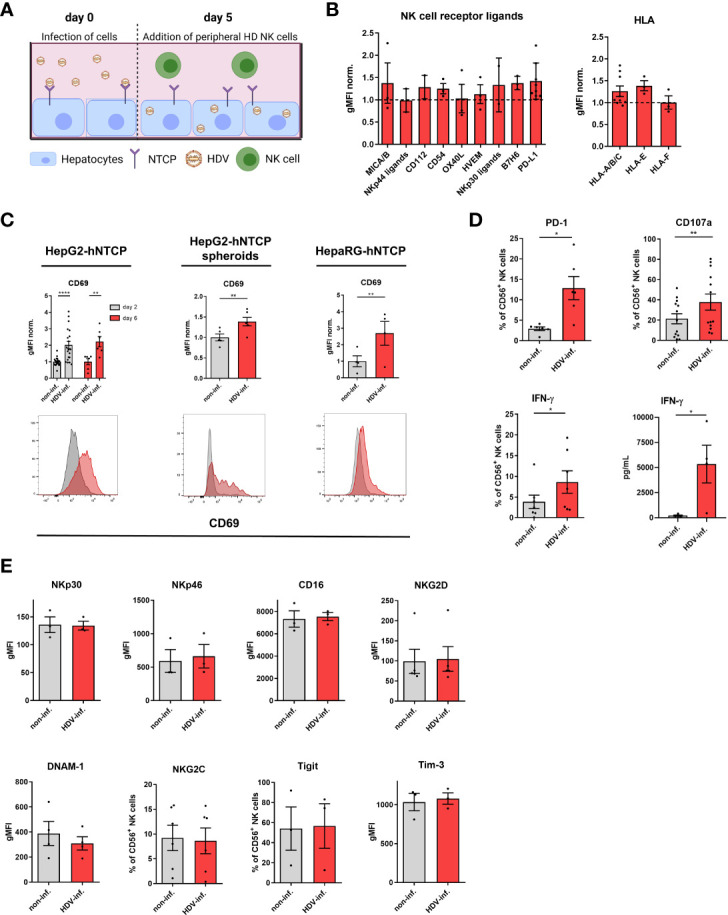
HDV-infected hepatocytes trigger NK cell activation. **(A)** Schematic overview of the established *in vitro* co-culture system of NK cells and HDV-infected hepatocytes (created with BioRender.com). **(B)** Surface expression of the NK cell receptor ligands MICA/B (n=3), NKp30 ligand (n=2), NKp44 ligand (n=2), CD54 (n=3), CD112 (n=2), OX40L (n=3), HVEM (n=3), B7H6 (n=3), PD-L1 (n=8) and of the HLA-A/B/C (n=9), HLA-E and HLA-F (n=3) molecules by HDV-infected HepG2-hNTCP cells 5 days after infection with HDV (bars represent the geometric mean of the fluorescence intensity normalized to the mean of non-infected controls +/- SEM). **(C)** Surface expression of CD69 on NK cells after co-culture with non-infected or HDV-infected HepG2-hNTCP cells for 2 or 6 days [n=6 (day6), n=20 (day2)], HepG2-hNTCP spheroids for 2 days (n=5) or HepaRG-hNTCP cells for 2 days (n=4) (bars represent the geometric mean fluorescence intensity of positive cells normalized to the mean of non-infected controls +/- SEM). Representative stainings are shown as histograms (grey: non-infected; red: HDV-infected). **(D)** Surface expression of PD-1 (n=6), CD107a (n=13), intracellular expression of IFN-γ (n=7) by NK cells and level of IFN-γ (n=4) in supernatants of co-cultures of NK cells with non-infected or HDV-infected HepG2-hNTCP cells after 2 days as determined by FACS analysis or Legendplex assay respectively (bars represent the mean frequency of positive cells or absolute amount of cytokines +/- SEM). **(E)** Surface expression of the activating receptors NKp30, NKp46, CD16, DNAM-1, and NKG2D (n=3) on NK cells among PBMCs or on isolated NK cells [NKG2C (n=6)] in co-culture with non-infected or HDV-infected HepG2-hNTCP cells after 2 days of co-culture and surface expression of the inhibitory receptors Tigit and Tim-3 on NK cells in co-culture with non-infected or HDV-infected HepG2-hNTCP cells after 2 days (n=3) (bars represent the mean geometric fluorescence intensity or frequency of positive cells +/- SEM). Levels of significance: *p<0.05, **p<0.01, ****p<0.0001 (student’s t-test).

To investigate the function of NK cells in HDV infection, we established a co-culture model using human NK cells and hepatocyte cell lines that overexpress the human HBV/HDV entry receptor hNTCP (**Figure 1A**). 5 days post infection of HepG2-hNTCP hepatoma cells with HDV, approx. 10% of the cells displayed an intracellular expression of the hepatitis D antigen as determined by flow cytometry and fluorescent immunohistochemistry staining (**Supplementary Figure 1A**). HDV infection did not significantly change the cell surface expression of the NK cell ligands MICA/B, NKp30/44 ligands, CD54, CD112, OX40L, HVEM, B7H6 and PD-L1 or the expression of HLA molecules on infected hepatoma cells (**Figure 1B**). To determine the NK cell response on hepatic cell lines after HDV infection, we assessed the expression of different cell surface markers, including CD69 after 2 and 6 days of co-culture (**Figure 1C**). NK cells displayed an increase in CD69, a marker for NK cell activation, both after 2 and 6 days of co-culture with HDV-infected HepG2-hNTCP cells compared to non-infected controls (**Figure 1C**). In addition to 2D co-cultures, we used 3D HepG2- hNTCP spheroids, whose structure and metabolism is considered to be of high physiological relevance (27). In concordance with the data obtained with 2D co-cultures, upon HDV-infection, spheroid co-cultured NK cells demonstrated an increase in CD69 expression (**Figure 1C**). In addition to CD69, NK cells from co-cultures with HDV-infected HepG2-hNTCP cells showed an upregulation of additional activation markers (**Figure 1D**). Programmed cell death protein (PD)-1, a molecule upregulated by activated/exhausted CD8^+^ T cells during viral infection (28–30), IFN-γ, which we detected by both intracellular staining and analysis of the cell culture SP and the degranulation marker CD107a were higher expressed by NK cells after encounter of HDV-infected cells (**Figure 1D**). Of note, expression of the activating receptors natural killer protein (NKp)30, NKp46, CD16, DNAX accessory molecule 1 (DNAM-1) and natural killer group (NKG)-2D (**Figure 1E**), and of the inhibitory receptors TIGIT and Tim-3 (**Figure 1E**) remained unchanged. In addition, the expression frequency of NKG2C, a marker of adaptive, memory-like NK cells, was not altered (**Figure 1E**). Furthermore, co-culture with HDV-infected cells did not alter the expression of Ki-67, indicative of cell proliferation, and the expression of the interleukin 2 receptor α chain (CD25) on NK cells (**Supplementary Figure 1B**). Taken together, our findings demonstrate that NK cells respond to HDV-infected hepatic cell lines, resulting in increased expression of activation markers.

### 3.2 NK cells eliminate HDV-infected hepatic cell lines via the TRAIL-TRAIL-R2 axis

**Figure 2:**
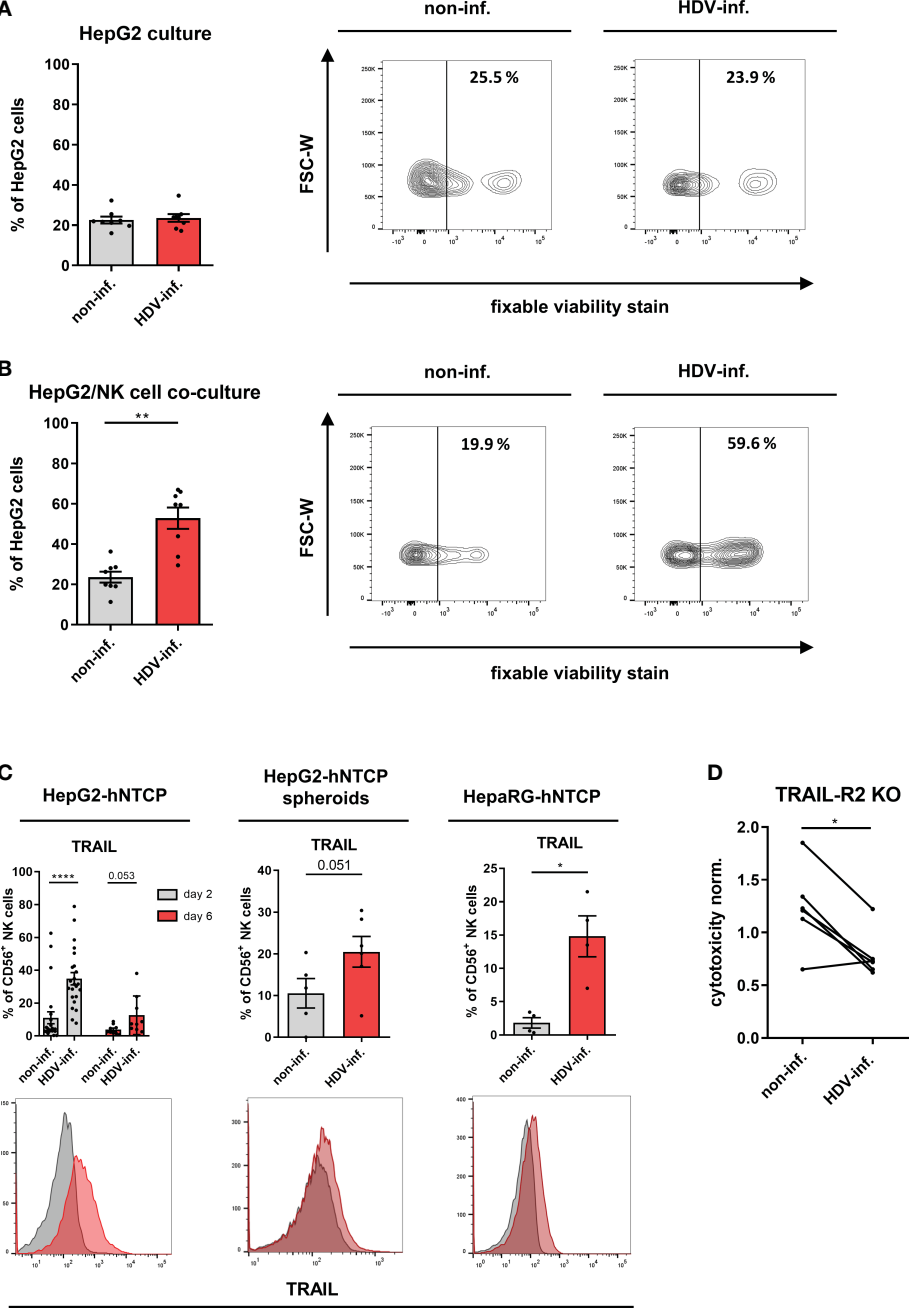
Cytotoxicity of NK cells in HDV infection. **(A)** Frequency of dead cells (fixable viability dye^+^) among non-inf. or HDV-inf. HepG2-hNTCP cells 7 days after infection with representative contour plots (n=8) (bars represent the mean frequency of positive cells +/- SEM). **(B)** Frequency of dead cells (fixable viability dye^+^) among non-inf. or HDV-inf. HepG2-hNTCP cells after 2 days of co-culture with NK cells with representative contour plots (n=8) (bars represent the mean frequency of positive cells +/- SEM). **(C)** Surface expression of TRAIL on NK cells after co-culture with non-infected or HDV-infected HepG2-hNTCP cells for 2 or 6 days [n=9 (day2), n=23 (day6)], HepG2-hNTCP spheroids for 2 days (n=6) or HepaRG-hNTCP cells for 2 days (n=4) (bars represent the geometric mean fluorescence intensity of positive cells normalized to the mean of non-infected controls +/- SEM). Representative stainings are shown as histograms (grey: non-infected; red: HDV-infected). **(D)** Cytotoxicity of NK cells against non-infected or HDV-infected HepG2-hNTCP-TRAILR2 knock out cells (n=6) as determined by LDH assay. Data is normalized to non-infected or HDV-infected HepG2-hNTCP-Cas9 control cells. Levels of significance: *p<0.05, **p<0.01, ****p<0.0001 (student’s t-test).

We next determined the cytolytic NK cell response in our co-cultures by determining the frequency of dead HepG2-hNTCP cells with impaired membrane integrity (fixable viability dye^+^). While HDV infection by itself did not induce cell death (**Figure 2A**), NK cells demonstrated a strong cytotoxicity towards HDV-infected HepG2-hNTCP cells (**Figure 2B**). Additionally, we observed preferential killing of HepG2-hNTCP cells that stained positive for the HDVAg by NK cells (**Supplementary Figure 2**).

To determine molecular pathways enriched in NK cells upon HDV infection, we compared the NK cell transcriptome after co-culture with non-infected or HDV-infected HepG2-hNTCP cells. Expression of transcripts encoding for the death receptor ligands FasL and TRAIL (TNFRSF10), and cytolytic effector molecules including granzyme B and perforin, were enriched (**Supplementary Figure 3A**), whereas other transcripts such as ones encoding for certain NK receptors in the “NK cell mediated cytotoxicity pathway” were downregulated for NK cells from HDV-infected cultures compared to non-infected cultures (**Supplementary Figure 3B**). In concordance with the transcriptomic data, we observed elevated protein expression of TRAIL on the NK cell surface in co-culture with HDV-infected HepG2-hNTCP or HepaRG-hNTCP cells (**Figure 2C**). Of note, TRAIL-R2 expression did not differ between HDVAg^-^ and HDVAg^+^ cells (**Supplementary Figure 3C**). Although upregulated at the transcriptional level, granzyme B and perforin protein expression by NK cells remained unchanged in the presence of HDV-infected cells (**Supplementary Figure 3D**). In addition, FasL surface expression was not detectable on co-cultured NK cells and tumor necrosis factor-α secretion by NK cells was not increased upon co-culture with HDV-infected hepatoma cells (data not shown). Both TRAIL-R1 and 2 are known to induce cell death upon ligand binding. Since TRAIL-R1 was only detectable to negligible amounts on HepG2 cells, we focused on TRAIL-R2 which was ubiquitously expressed (**Supplementary Figure 4A**). To determine whether in HDV-infected co-cultures NK cells kill target cells via TRAIL-TRAIL-R2 interaction, we generated HepG2-hNTCP cells deficient in TRAIL-R2 expression (hNTCP-TR2 KO) (**Supplementary Figure 4A**). Expression of the HDV entry receptor NTCP, frequency of infected cells and the expression of the surface molecules MHC class I chain related-proteins A and B (MICA/B), Tumor necrosis factor receptor (TNFR)1 and CD155 that potentially regulate NK cell cytotoxicity did not differ between control cells and hNTCP-TR2 KO cells (**Supplementary Figures 4B–D**). Upon NK cell co-culture, we observed that HDV-infected TRAIL-R2 KO HepG2-hNTCP cells were less susceptible to NK cell-mediated cytotoxicity compared to HDV-infected control targets (**Figure 2D**). Of note, the expression of TRAIL-R1/2 and the decoy receptors TRAIL-R3/4 was not altered or induced upon infection with HDV, respectively (**Supplementary Figure 4E**). Peripheral blood NK cells can be divided into CD56^bright^ NK cells, which are considered to mainly produce cytokines and to be precursors to CD56^dim^ NK cells, which are considered to mainly exert cytotoxicity (31). In presence of HDV-infected cells, CD56^bright^ NK cells displayed higher levels of IFN-γ, PD-1, CD107a, CD69 and TRAIL (**Supplementary Figure 5**). Collectively, these data demonstrate that in HDV-infected co-cultures NK cells upregulate TRAIL expression and kill HDV-infected hepatic cell lines using the TRAIL/TRAILR2 pathway.

### 3.3 Activated NK cells do not inhibit a T cell response in HDV infection *in vitro*

**Figure 3:**
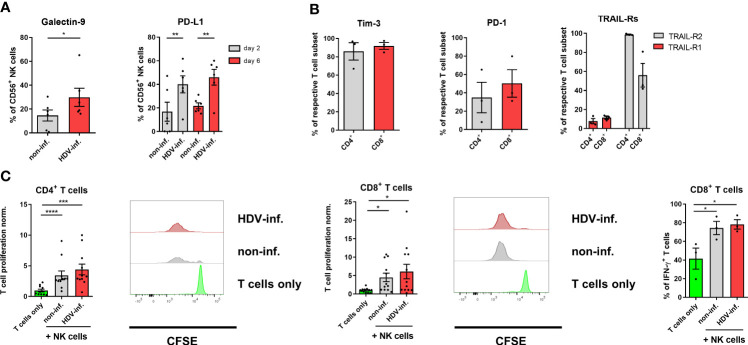
Influence of NK cells on T cells in HDV infection. **(A)** Surface expression of Galectin-9 and PD-L1 on NK cells after 2 days or 2 and 6 days of co-culture with non-infected or HDV-infected HepG2-hNTCP cells, respectively (n=6) (bars represent the mean frequency of positive cells +/- SEM). **(B)** Surface expression of Tim-3, PD-1 and TRAIL-R1/2 on CD3/CD28-stimulated CD4^+^ and CD8^+^ T cells (n=3) (bars represent the mean frequency of positive cells +/- SEM). **(C)** Proliferation of CD3/CD28 stimulated CD4^+^ and CD8^+^ T cells (n=11) and frequency of IFN-γ production (n=3) of CD8^+^ T cells alone, or in the presence of NK cells from co-cultures with non or HDV-infected HepG2-hNTCP cells after 96 h with representative histograms. NK cell/T cell ratio (1:4) (bars represent the mean frequency of positive cells normalized to the T cell only control +/- SEM or mean frequency of positive cells +/- SEM). Levels of significance: *p<0.05, **p<0.01, ***p<0.001, ****p<0.0001 (student’s t-test).

NK cells were previously described to negatively regulate the activity of T cells in pathological conditions. It was shown that this interplay can be mediated by Galectin-9, programmed death-ligand (PD-L)1 or TRAIL on NK cells and the corresponding receptors Tim-3, PD-1 and TRAIL-R1/2 on T cells (13, 32, 33). Since we observed an upregulation of Galectin-9, PD-L1 and TRAIL expression frequency on NK cells in co-culture with HDV-infected cells (**Figures 2C**, **3A**), and Tim-3, PD-1 and TRAIL-Rs on CD3/CD28-stimulated autologous T cells (**Figure 3B**), we determined if NK cells from our co-culture system differentially affect the proliferation assessed by CFSE dilution and IFN-γ production by autologous T cells. NK cells isolated from co-cultures with non-infected HepG2-hNTCP cells, as well as from co-cultures with HDV-infected cells, promoted proliferation of activated CD4^+^ T helper and cytotoxic CD8^+^ T cells (**Figure 3C**). In addition, NK cells isolated from both HDV-infected or non-infected co-cultures induced a similar intracellular IFN-γ production by CD8^+^ T cells (**Figure 3C**). Altogether, this data reveals that NK cells, conditioned by HDV-infected hepatoma cells, did not inhibit autologous T cells *in vitro*.

### 3.4 IFN-β induces NK cell activation in HDV infection

**Figure 4:**
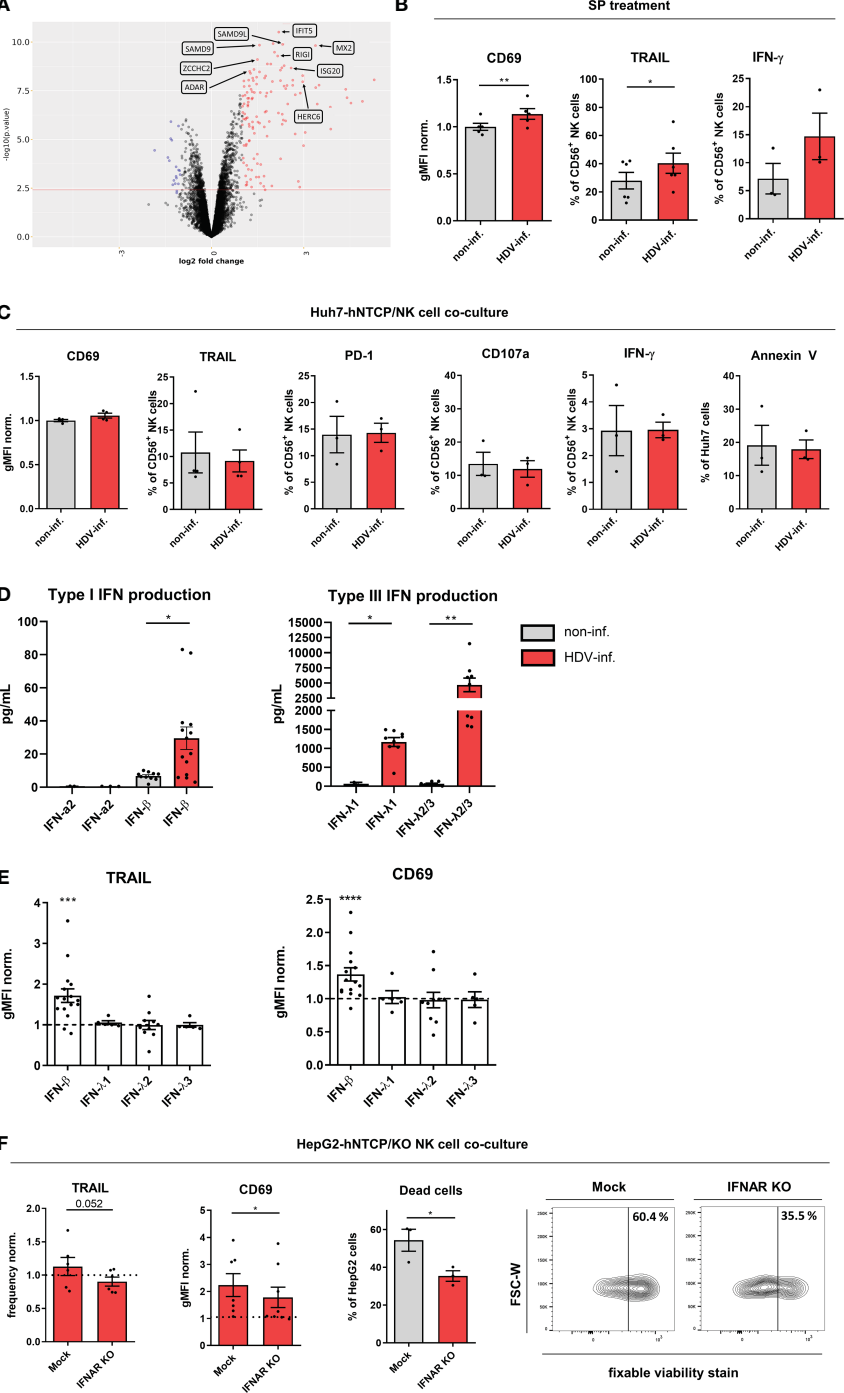
IFN-β induces NK cell activity in HDV infection. **(A)** Volcano plot of differentially expressed genes (red: upregulated; blue: downregulated) by peripheral healthy donor NK cells in co-culture with non-infected or HDV-infected HepG2-hNTCP cells (n=5). **(B)** CD69, TRAIL surface (n=4) and intracellular IFN-γ (n=3) expression on NK cells treated with supernatants (SP) from non-infected or HDV-infected HepG2-hNTCP cells for 24h (bars represent the geometric mean fluorescence intensity of positive cells normalized to the mean of non-infected controls +/- SEM or mean frequency of positive cells +/- SEM). **(C)** Surface expression of CD69 and TRAIL (n=4), PD-1 and CD107a (n=3), intracellular expression of IFN-γ (n=3) by NK cells after 2 days of co-culture with non-infected or HDV-infected Huh7-hNTCP cells and frequency of apoptotic cells (Annexin V^+^) among non-infected or HDV-infected Huh7-hNTCP cells in co-culture with NK cells after 2 days (n=3) (bars represent the geometric mean fluorescence intensity of positive cells normalized to the mean of non-infected controls +/- SEM or mean frequency of positive cells +/- SEM). **(D)** Levels of IFN-α2 (n=2-3), IFN-β (n=10-14), IFN-λ1 (n=2-9) and IFN-λ2/3 (n=7-9) in cell culture supernatants of non-infected or HDV-infected HepG2-hNTCP cells 2 days after infection (bars represent the absolute amount of cytokines +/- SEM). **(E)** Surface expression of TRAIL or CD69 by CD56^+^ NK cells after treatment with either IFN-β (n=16); λ−1 (n=5)γ λ−2 (n=10)γ λ−3 (n=5) (2ng/mL) for 24h (n=19) (bars represent the geometric mean fluorescence intensity of positive cells normalized to the mean of untreated controls +/- SEM). **(F)** TRAIL (n=6) or CD69 (n=7-8) surface expression of CD56^+^ NK mock control and IFNαβR KO cells and frequency of dead (fixable viability^+^) HepG2-hNTCP cells after 48h of co-culture cells with representative contour plots (n=3) (bars represent the mean frequency of positive cells or the mean frequency or geometric mean fluorescence intensity of positive cells normalized to the mean of non-infected controls +/- SEM). *p<0.05, **p<0.01, ***p<0.001, ****p<0.0001 (student’s t-test).

Among differentially expressed genes, the ISGs including ADAR, ZCCHC2, SAMD9/SAMD9L, IFIT5, RIG1, MX2, ISG20 and HERC6, which have all been described to be involved in the innate anti-viral response, were among the most significantly enriched in NK cells from HDV-infected co-cultures compared to non-infected cultures (**Figure 4A**) (34–40). In addition, we observed downregulation of the ubiquitin-activating enzymes UBE2K and UBE2G2 (**Supplementary Figure 6A**). Furthermore, SP of HDV-infected HepG2-hNTCP cells was able to increase CD69 and TRAIL and intracellular IFN-γ expression (**Figure 4B**). In addition, Huh-7-hNTCP cells, which have been recently shown to be unable to mount an IFN response (41–43), did not induce the activation of NK cells and subsequently did not show increased susceptibility to NK cell-mediated cytotoxicity upon HDV infection (**Figure 4C**).

We therefore analyzed the production of type I interferons IFN-α, β and type III interferons Λ-1/2/3 by HepG2-hNTCP cells upon HDV infection as soluble mediators of NK cell activation. We detected both an increase in IFN-β and IFN-Λ-l/2/3 in SPs of HepG2-hNTCP cells in response to HDV infection (**Figure 4D**). Among these factors, only IFN-β was able to mediate an increase in TRAIL and CD69 expression on NK cells *in vitro* (**Figure 4E**). In addition, the majority of transcripts associated with the IFN-β pathway were also more abundant in NK cells upon co-culture with HDV-infected cells compared to NK cells from non-infected co-cultures (**Supplementary Figure 6A**). To confirm the role of IFN-β in mediating the observed NK cell activation upon encounter of HDV-infected cells *in vitro*, we generated NK cells deficient for the receptor for IFN-β, IFNAR. These IFNAR-KO NK cells showed a reduced phosphorylation of the IFNAR downstream target signal transducer and activator of transcription (STAT)2 and a reduced expression of TRAIL after *in vitro* stimulation with IFN-β (**Supplementary Figure 6B**). More importantly, these cells displayed a reduced TRAIL and CD69 induction in the presence of HDV-infected cells and impaired cytotoxicity towards these cells (**Figure 4F**). In summary, these findings highlight IFN-β as an important factor mediating NK cell activation in HDV infections.

### 3.5 NK cell phenotype in HBV/HDV patients

**Figure 5:**
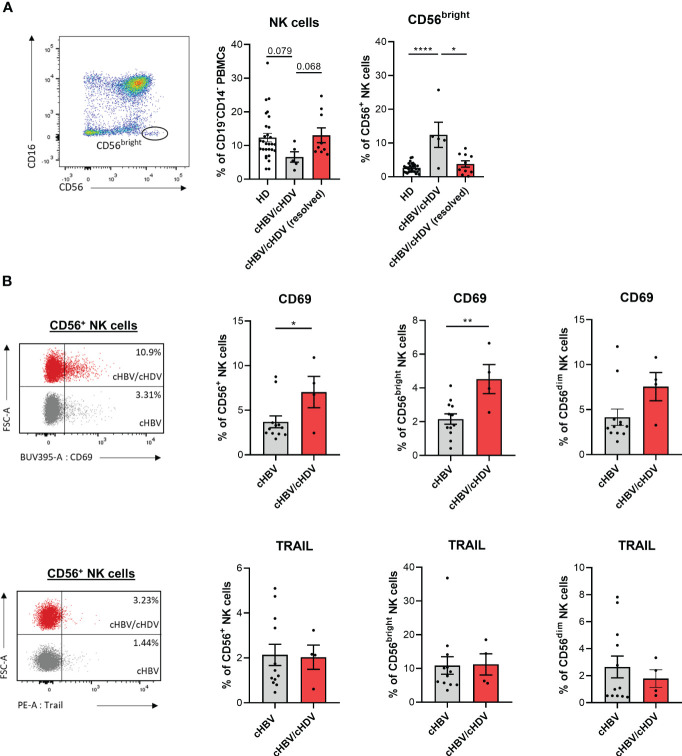
NK cell phenotype in hepatitis patients. **(A)** Frequency of circulating CD3^-^CD16^+^CD56^dim^ and CD3^-^CD16^-^CD56^bright^ NK cells in healthy donors (HD) (n=29), chronic hepatitis B/D (cHBV/cHDV) (n=5) and resolved cHBV/cHDV patients among PBMCs (n=9) (bars represent the mean frequency of cells +/- SEM). **(B)** Expression of CD69 or TRAIL on circulating CD3^-^CD16^+^CD56^dim^ and CD3^-^CD16^-^ CD56^bright^ NK cells in cHBV (n=12) and cHBV/cHDV (n=4) patients (bars represent the mean frequency of positive cells +/- SEM). Levels of significance: *p<0.05, **p<0.01, ****p<0.0001 (student’s t-test).

To investigate the clinical relevance of our findings, we analyzed the frequencies of NK cells in peripheral blood and their phenotype in HDs, cHBV and cHBV/cHDV patients. The patient cohort was almost exclusively cytomegalovirus (CMV) positive making an influence of the CMV status on our analysis unlikely (**Supplementary Tables 1–5**). The frequency of peripheral NK cells among PBMCs was decreased by HDV infection which was accompanied by a shift towards the CD16^-^CD56^bright^ compartment (**Figure 5A**). In patients with resolved cHDV infection (PCR negative), both the frequency of total NK cells and of CD56^bright^ NK cells were comparable to HDs (**Figure 5A**). In our cohort of cHBV and cHBV/cHDV patients, we observed upregulation of CD69, while expression of TRAIL remained unaffected (**Figure 5B**). In conclusion, cHDV infection causes a decrease in the frequency of peripheral NK cells and induces a shift of the NK cell population towards the CD56^bright^ NK cell compartment. In addition, the higher frequency of CD69 expression in cHBV/cHDV patients implies a higher activation potential of these cells compared to cHBV patients.

## 4 Discussion

Contemporaneous infection of hepatocytes with HBV is necessary for the generation of infectious HDV particles. Nevertheless, HDV is capable of infecting hepatocytes and intracellular replication independent of HBV co-infection. More recently, also proliferation of HDV-mono-infected cells was shown to represent an important mechanism of HDV spread in the liver (4). In our co-culture system, we focused on the interaction between NK cells and HDV mono-infected cells *in vitro*. In HDV-infected co-cultures, NK cells displayed higher CD69 and TRAIL expression and higher IFN-γ production compared to non-infected co-cultures, indicative of their activation. Using a CRISPR/Cas9-mediated gene deletion, we demonstrated the importance of TRAIL for NK cell-mediated killing of TRAIL-R2 expressing HDV-infected hepatoma cells. Cytotoxicity of NK cells towards infected cells is particularly important to limit viral spread in the early stages of infection (44). This corresponds to the rapid but transient expression of TRAIL on NK cells in HDV-infection that we observed. In addition, similar kinetics of TRAIL expression were seen in LCMV-infected mice (45). The lower expression of TRAIL by NK cells after longer co-culture with HDV-infected cells observed on day 6 of co-culture is likely due to the killing of IFN-β producing HDV-infected cells by NK cells as we did not detect IFN-β after 6 days of co-culture in supernatants. Kinetics of TRAIL expression may be also regulated by sheddases such as metalloproteinases 2, but studies on this issue are controversial and we did not address this topic in our study (46). Recent studies demonstrated that engagement of TRAIL on NK cells can contribute to their degranulation (47). This indicates that NK cells do not only kill HDV-infected cells directly by TRAIL but are also triggered to degranulate as an additional cytotoxic mechanism. This is supported by our observation of increased degranulation of NK cells in infected co-cultures, and the incomplete inhibition of killing by KO of TRAIL-R2. Accumulating evidence indicates that NK cells do not only fulfill a cytotoxic effector function towards infected or tumor cells, but can also exert an immunoregulatory function towards T cells (48). Despite these observations, NK cells co-cultured with HDV-infected cells did not inhibit but induced proliferation of CD3/CD28 activated T cells and IFN-γ production. Activation of T cells by NK cells can be mediated by cell-contact dependent interaction including the OX40-OX40L and CD28-B7 axis or by secretion of IL-2 by NK cells (49–52). We also observed a strong cytotoxicity of NK cells in the presence of HDV-infected cells and additionally, preferential killing of hepatoma cells expressing the HDV antigen. This observation was not caused by differential expression of TRAIL receptors on hepatic cell lines upon HDV infection. We therefore suggest that NK cells in close proximity to HDVAg^+^ cells, hence IFN-β producing cells, gain a high cytotoxic capacity which leads to the killing of adjacent HDVAg^+^ cells. Of note, HepG2-hNTCP cells express the NK cell modulating molecules CD155 and MICA/B, and as a hepatocellular carcinoma cell line, induce activation of NK cells also in the absence of HDV-infection. Although we observed some expression of NK cell activation markers in co-culture with non-infected hepatic cells and moderate NK cell-mediated killing of these cells, we detected much stronger NK cell responses against HDV-infected HepG2-hNTCP cells supporting the relevance of HepG2-hNTCP/NK cell co-culture systems as a useful tool to study the immune response against HDV *in vitro*.

Together, our observations suggest an increased activation of NK cells in the presence of HDV-infected cells which may contribute to the severe liver damage found in HDV patients. The data at hand indicates IFN-β production by infected cells as an important mechanism in NK cell activation. These results are in line with different studies demonstrating an increased production of type-I and type III interferons by hepatocytes upon HDV infection, dependent on the sensing of HDV-RNA by melanoma differentiation-associated protein 5 (4, 43, 53, 54). ISG RIG1, which we found to be upregulated in NK cells upon encounter of HDV-infected cells, has been described to be a potent inducer of TRAIL and to support NK cell cytotoxicity without changing the expression of NK cell receptors, which is in concordance with our findings (38). In summary, these data suggest RIG1 as a potential factor to regulate NK cell cytotoxicity in HDV infection. Of note, several genes associated with IFN-β signaling were downregulated in NK cells encountering HDV-infected cells, including the ubiquitin-conjugating enzymes UBE2K and UBE2G2. Since proteins of the UBE family have been shown to dampen the IFN response and to promote viral replication, their down-regulation may support the NK cell-mediated anti-viral response (55, 56).

We detected a decrease in the frequency of peripheral NK cells in cHBV/cHDV-infected individuals compared to HDs, which was restored upon successful therapy. Kefalakes and colleagues demonstrated a reduction of intrahepatic NK cells in HDV-infected patients compared to HD controls (57). Therefore, increased apoptosis or decreased repopulation of NK cells rather than a sequestration of NK cells to the liver may partially explain our observation.

In line with published data, showing that activated (HLA-DR^+^) NK cells were increased in HDV-infected patients compared to HDs, we observed an increase in CD69 expression frequency by NK cells from cHBV/cHDV-infected individuals (57). TRAIL expression on NK cells remained similar between HBV and HBV/HDV patients which could be due to non-detectable levels of type I IFNs in sera of HBV and HBV/HDV patients (data not shown). The lack of detectable type I IFNs in serum might be due to the fact that type I IFNs are primarily produced locally in the liver in hepatitis patients (58–60). The fact that we observed CD69 upregulation, but not TRAIL upregulation on peripheral NK cells in HDV patients, could be due to the observation, that CD69 upregulation can be mediated by other factors besides type I IFNs, including CD16 activation, IL-2 and direct activation of protein kinase C (61).

The shift we observed in the composition of the peripheral NK cell compartment towards CD56^bright^ NK cells was previously reported for HBV patients (62). In the same study, the frequency of peripheral CD56^bright^ TRAIL^+^ NK cells in HBV patients correlated with liver damage. The expansion of the CD56^bright^ compartment may be due to exhaustion and subsequent apoptosis of the CD56^dim^ compartment. The expansion of CD56^bright^ NK cells that we observed in non-resolved HDV patients may therefor partially explain the high degree of liver damage found in these patients (63). Taken together, the altered frequency of CD56^bright^ NK cells in patients together with their higher expression frequency of activation markers and TRAIL upon encounter of HDV-infected cells *in vitro* compared to CD56^dim^ cells indicate a prominent role of the CD56^bright^ compartment in the immune response against HDV. NK cells represent up to 50% of all intrahepatic lymphocytes. Although CD56^bright^ NK cells only account for 10% of circulating NK cells, they represent up to 50% of liver-resident NK cells, which further suggests that in particular CD56^bright^ NK cells might participate in the immune response against hepatotropic viruses (64, 65).

In summary, our study identifies the TRAIL-TRAIL-R2 axis as an important mechanism for NK cells to target HDV-infected cells. Moreover, our findings demonstrate IFN-β as a factor, released by infected hepatoma cells that activates NK cells. Although it needs to be considered that NK cells might also mediate liver damage, our data indicate a high potential for NK cells as effector cells for the clearance of HDV *in vivo*.

## Data availability statement

For the RNA-Seq the count data were transformed to TPM and FPKM expression values and the resulting count matrices are available via the link https://www.ncbi.nlm.nih.gov/geo/query/acc.cgi?acc=GSE249240 (GEO accession number: GSE249240). Requests to access other datasets for this article should be directed to the corresponding author.

## Ethics statement

The studies involving humans were approved by Ethik Kommission II, Medical Faculty Mannheim Ethik Kommission, Albert-Ludwigs-Universität Freiburg. The studies were conducted in accordance with the local legislation and institutional requirements. The human samples used in this study were acquired from gifted from another research group. Written informed consent for participation was not required from the participants or the participants’ legal guardians/next of kin in accordance with the national legislation and institutional requirements. For the isolation of primary NK and T cells buffy coats were provided by the DRK-Blutspendedienst (according to the principles of the Declaration of Helsinki). Prior to blood donation, written consent was obtained by all donors (approved by the Ethik Kommission II of the Medical Faculty Mannheim, 87/04). Frozen PBMC of HBV, HDV/HBV patients and HDs for flow cytometric analysis of NK cells and analysis of cytokines were provided by the Freeze Biobank, University Hospital Freiburg (project number: 383/19, project number: 512/19 (project manager: CN-H), project number: 515/19 (project manager: Dr. Maike Hofmann) (**Supplementary Tables 5–9**). Prior to sample acquisition, all individuals provided written informed consent.

## Author contributions

CG: Conceptualization, Data curation, Formal Analysis, Methodology, Visualization, Writing – original draft, Writing – review & editing. JM: Conceptualization, Data curation, Formal Analysis, Methodology, Visualization, Writing – review & editing. IG: Conceptualization, Data curation, Formal Analysis, Methodology, Validation, Writing – review & editing. TH: Data curation, Formal Analysis, Methodology, Writing – review & editing. ZZ: Conceptualization, Methodology, Resources, Supervision, Writing – review & editing. YN: Conceptualization, Formal Analysis, Methodology, Resources, Supervision, Writing – review & editing. FK: Data curation, Resources, Writing – review & editing. MH: Conceptualization, Supervision, Writing – review & editing. CN-H: Conceptualization, Supervision, Writing – review & editing. CS: Data curation, Formal Analysis, Methodology, Resources, Software, Visualization, Writing – review & editing. KR: Methodology, Resources, Software, Writing – review & editing. SU: Conceptualization, Funding acquisition, Methodology, Resources, Supervision, Writing – review & editing. AC: Funding acquisition, Methodology, Project administration, Resources, Supervision, Writing – review & editing. IS: Data curation, Formal Analysis, Methodology, Writing – review & editing.

## Glossary

**Table:**

(c)HBV	(chronic) hepatitis B virus
(c)HCV	(chronic) hepatitis C virus
(c)HDV	(chronic) hepatitis D virus
(h)NTCP	(human) sodium taurocholate co-transporting polypeptide
BME	basement membrane extract
CFSE	carboxyfluorescein succinimidyl ester
CMV	cytomegalovirus
crRNA	clustered regularly interspaced short palindromic repeats RNAs
DMSO	dimethyl sulfoxide
DNAM-1	DNAX accessory molecule 1
E:T	effector:target
FBS	fetal bovine serum
gMFI	geometric mean fluorescence intensity
HD	healthy donor
HDAg	hepatitis D antigen
HLA	human leukocyte antigen
HLADR	human leukocyte antigen -DR isotype
IFN	interferon
IL	interleukin
ISG	interferon-stimulated gene
KO	knock out
LDH	lactate dehydrogenase
MICA/B	MHC class I chain related-proteins A and B
NK cell	natural killer cell
NKG	natural killer group
NKp	natural killer protein
P/S	penicillin/streptomycin
PBS	phosphate-buffered saline
PD-1	programmed cell death protein 1
PD-L1	programmed death-ligand 1
PEG	polyethylene glycol
PFA	paraformaldehyde
pSTAT2	phosphorylated signal transducer and activator of transcription 2
SEM	standard error of the mean
sgRNA	single guide RNA
SP	supernatant
TNFR	tumor necrosis factor receptor
tracrRNA	trans-activating crispr RNA
TRAIL	tumor necrosis factor related apoptosis inducing ligand
Com	co-morbidity
GOT	glutamat-oxalacetat-transaminase
GPT	glutamate-pyruvatetransaminase
n.a.	not available
NUC	nucleos(t)ide analogs
rHAV	resolved hepatitis A virus
rHDV	resolved hepatitis D virus
trHBV	treatment resolved hepatitis B virus
trHDV	treatment hepatitis D virus
