# Supplementary material for: Hepatitis D infection induces IFN-β-mediated NK cell activation and TRAIL-dependent cytotoxicity
Source: Front Immunol. 2023 Dec 8;14:1287367. doi: 10.3389/fimmu.2023.1287367 (PMC10739304; doi:10.3389/fimmu.2023.1287367)
Supplement: Supplementary file 1 [file DataSheet_1.pdf]

Supplementary Material

1. Supplementary tables

Supplementary table 1: Commercial antibodies used for flow cytometry

Antigen	Fluorophore	Clone	Company	Cat.:	Isotype
B7H6	PE	875001	R&D Systems	FAB7144P	Mouse IgG1, κ
CD3	APC	UCHT1	Biolegend	300458	Mouse IgG1, κ
CD3	BV786	UCHT1	BD	565491	Mouse IgG1, κ
CD3	FITC	UCHT1	BD	300440	Mouse IgG1, κ
CD3	AlexaFluor594	UCHT1	BioLegend	300446	Mouse IgG1, κ
CD4	PerCP	SK3	BD	345770	Mouse IgG1, κ
CD4	PE	RPA-T4	Biolegend	300507	Mouse IgG1, κ
CD8a	PE-Cy7	SK1	Biolegend	344712	Mouse IgG1, κ
CD8a	APC	SK1	Biolegend	980904	Mouse IgG1, κ
CD14	APC-eF780	61D3	eBioscience	47-0149-42	Mouse IgG1, κ
CD16	PerCP-Cy5.5	3G8	Biolegend	302028	Mouse IgG1, κ
CD16	V500	3G8	BD	561394	Mouse IgG1, κ
CD19	APC-eF780	HiB19	eBioscience	47-0199	Mouse IgG1, κ
CD25	BV650	BC96	Biolegend	302633	Mouse IgG1, κ
CD54	PE	HCD54	Biolegend	322708	Mouse IgG1, κ
CD56	BV605	5.1H11	Biolegend	362538	Mouse IgG1, κ
CD56	PE-Cy7	HCD56	Biolegend	318318	Mouse IgG1, κ
CD56	BV650	5.1H11	Biolegend	362532	Mouse IgG1, κ
CD69	BV421	FN50	Biolegend	310929	Mouse IgG1, κ
CD69	BV711	FN50	BD	563836	Mouse IgG1, κ
CD69	BV395	FN50	BD	564364	Mouse IgG1, κ
CD107a	FITC	H4A3	Biolegend	328606	Mouse IgG1, κ
CD112	PE-Cy7	TX31	Biolegend	337413	Mouse IgG1, κ
DNAM-1	FITC	11A8	Biolegend	338303	Mouse IgG1, κ
CD155	PE	SKII.4	Biolegend	337609	Mouse IgG1, κ
FasL	BV421	NOK-1	Biolegend	306411	Mouse IgG1, κ
Galectin-9	PE-Cy7	9M1-3	Biolegend	348915	Mouse IgG1, κ
Granzyme B	Pacific Blue	GB11	Biolegend	515408	Mouse IgG1, κ
HLA-A/B/C	BV605	W6/32	Biolegend	311431	Mouse gG2a,κ
HLA-E	PE	3D12	Biolegend	373203	Mouse IgG1, κ
HLA-F	PE	3D11	Biolegend	373203	Mouse IgG1, κ
HVEM	PE	122	Biolegend	318805	Mouse IgG1, κ
IFN-g	APC	B27	BD	554702	Mouse IgG1, κ
IFN-g	BV605	4S.B3	Biolegend	502535	Mouse IgG1, κ
IFN-g	PE	B27	Biolegend	506507	Mouse IgG1, κ
Ki-67	PE	Ki-67	Biolegend	350504	Mouse IgG1, κ
Ki-67	BV421	Ki-67	Biolegend	350506	Mouse IgG1, κ
MICA/B	PE	6D4	Biolegend	320906	Mouse IgG2b, κ
NKp30	BV605	P30-15	BD	563384	Mouse IgG1, κ
NKp46	APC	9E2/Nkp46	BD	558051	Mouse IgG1, κ
NKG2C	PE	FAB138P	R&D Systems	134591	Mouse IgG1, κ
NKG2D	BV605	1D11	Biolegend	320831	Mouse IgG1, κ
OX40L	PE	11C3.1	Biolegend	326308	Mouse IgG1, κ
PD-1	BV421	NAT105	Biolegend	367421	Mouse IgG1, κ
PD-1	FITC	MIH43	eBioscience	11-9969-41	Mouse IgG1, κ
PD-1	PerCP-Cy5.5	EH12	BD	561273	Mouse IgG1, κ
PD-L1	PE	MIH1	BD	557924	Mouse IgG1, κ
PD-L1	PE-Cy7	29E.2A3	Biolegend	329717	Mouse IgG2b, κ
PD-L1	PE-CF594	MIH1	BD	563742	Mouse IgG1, κ

Perforin	PE	B-D48	Biolegend	353304	Mouse IgG1, κ
pSTAT2	FITC	polyclonal	ThermoFisher	PA5-46888	Rabbit / IgG
Tim-3	PerCP-Cy5.5	F38-2E2	Biolegend	345015	Mouse IgG1, κ
Tim-3	BV421	F38-2E2	Biolegend	345002	Mouse IgG1, κ
TNF-R1	APC	W15099A	Biolegend	369905	Mouse IgG2a, κ
TRAIL	APC	RIK-2	Biolegend	308209	Mouse IgG1, κ
TRAIL	PE-Cy7	RIK-2	Biolegend	308216	Mouse IgG1, κ
TRAIL	PE	RIK-2	Biolegend	308206	Mouse IgG1, κ
TRAIL	PE	RIK-2	BD	550516	Mouse IgG1, κ
TRAIL-R1	PE-Cy7	DJR1	Biolegend	307209	Mouse IgG1, κ
TRAIL-R1	APC	DJR1	Biolegend	307207	Mouse IgG1, κ
TRAIL-R2	PE	DJR2-4 (7-8)	Biolegend	307405	Mouse IgG1, κ
TRAIL-R3	APC	DJR3	Miltenyi	130-104-788	Mouse IgG1, κ
TRAIL-R4	FITC	TRAIL-R4-01	ThermoFisher	A15752	Mouse IgG1, κ

Supplementary table 2: Antibodies used as isotype controls

Fluorophore	Clone	Company	Cat.:	Isotype
APC	MOPC-21	Biolegend	400120	Mouse IgG1, κ
PE-Cy7	MOPC-21	Biolegend	400126	Mouse IgG1, κ
PE	MOPC-21	Biolegend	400112	Mouse IgG1, κ
BV605	X40	BD	562652	Mouse IgG1, κ
Pacific Blue	MOPC-21	Biolegend	400131	Mouse IgG1, κ
PE-Cy7	MPC-11	Biolegend	400325	Mouse IgG2b, κ
PE-CF594	X40	BD	562292	Mouse IgG1, κ
BV421	MOPC-21	Biolegend	400158	Mouse IgG1, κ
FITC	MOPC-21	Biolegend	554679	Mouse IgG1, κ
PerCP-Cy5.5	MOPC-21	Biolegend	400150	Mouse IgG1, κ
BV711	MOPC-21	Biolegend	400168	Mouse IgG1, κ
BV786	X40	BD	563330	Mouse IgG1, κ
PerCP	MOPC-21	Biolegend	400147	Mouse IgG1, κ
BV650	MOPC-21	BD	400163	Mouse IgG1, κ
PE	MOPC-173	Biolegend	400214	Mouse IgG2a, κ
BV605	MOPC-173	Biolegend	400269	Mouse IgG2a, κ
APC	MOPC-173	Biolegend	400221	Mouse IgG2a, κ
FITC	polyclonal	ThermoFisher	11-4614-80	Rabbit / IgG

Supplementary table 3: Recombinant human chimera proteins

	Company	Cat.:	Isotype
NKp30-Fc	R&D Systems	1849-NK	human IgG1
NKp44-Fc	R&D Systems	2249-NK	human IgG1
Human IgG1-Fc	R&D Systems	110-HG	human IgG1

Supplementary table 4: Secondary antibodies

Antigen	Fluorophore	Clone	Company	Cat.:
Human IgG (H+L)	PE	Polyclonal	Jackson ImmunoResearch	109-116-170

Supplementary table 5: Healthy donors

Gender	Age	CMV status
f	28	+
f	26	+
f	27	+
f	82	+
f	24	+
f	31	+
m	23	+
f	22	+
m	33	+
f	27	+
f	28	+
f	25	+
f	29	+
f	25	+
f	45	+
m	54	+
m	27	+
m	34	+
f	31	-
f	26	-
f	21	-
f	25	-
f	26	-
f	29	-
m	20	-
m	25	-
m	23	-
f	22	-
f	28	-
	31.3 (Mean)	

Supplementary table 6: cHBV/cHDV patient cohort

Diagnose	Gender	HBV-PCR [IU/ml]	Anti-HDAg IgG	HDV-PCR [IU/ml]	Therapy	Age	GOT [U/l]	GPT [U/l]	HBeAg	clinical phase	CMV status
cHBV/cHDV/rHAV/Com	f	<10	+	688002	Viread (since 2017)	32	67	133	-	HBeAg- chronic hepatitis B	+
cHBV / cHDV	f	<10	+	18762	naive, low viral load, low ALT/AST	37	33	36	-	HBeAg- chronic HBV infection	+
cHBV/cHDV	f	14052	+	100000	Nuc	51	63	58	+	HBeAg+/(NUC-related) chronic hepatitis B	+
cHBV/cHDV	m	453	+	316000	naive	38	43	36	n.a.	n.a.	+
cHBV/cHDV	m	110	+	107627	status post PEG-IFN	34	142	324	n.a.	n.a.	+
						38.4 (Mean)					

Supplementary table 7: cHBV/cHDV (resolved) patient cohort

Diagnose	Gender	HBV-PCR [IU/ml]	Anti-HDAg IgG	HDV-PCR [IU/ml]	Therapy	Age	GOT [U/l]	GPT [U/l]	HBeAg	clinical phase	CMV status
cHBV/rHDV	f	350	+	0	naive, low viral load, low ALT/AST	33	23	23	-	HBeAg- chronic HBV infection	+
cHBV/rHDV	f	134	+	0	naive	43	22	26	n.a.	n.a.	+
cHBV/rHDV	f	80	+	0	naive, low viral load, low ALT/AST	28	59	84	-	HBeAg- chronic HBV infection	+
trHBV/trHDV	m	0	+	0	Entecavir	60	24	29	+	HBeAg+/(NUC-related) chronic hepatitis B	+
trHBV/trHDV	m	0	+	0	status post PEG-IFN	38	20	13	n.a.	n.a.	+
trHBV/rHCV/trHDV	m	0	+	0	status post PEG-IFN	51	39	34	-	HBeAg- chronic hepatitis B	+
cHBV/rHCV/rHDV	m	6593	+	0	naive	37	n.a.	38	n.a.	n.a.	+
cHBV/rHDV	m	10	+	0	naive, low viral load, low ALT/AST	53	22	22	-	HBeAg- chronic HBV infection	-
cHBV/trHDV	m	10	+	0	Tenofovir (since 2014)	29	74	84	+	HBeAg+/(NUC-related) chronic hepatitis B	-
						41.3 (Mean)					

Supplementary table 8: cHBV patient cohort for TRAIL and CD69 analysis

Diagnose	Gender	HBV-PCR [IU/ml]	Therapy	Age	GOT [U/I]	GPT [U/I]	HBeAg+	clinic phase	CMV status
cHBV	m	0	Entecavir (since 2009)	37	21	36	-	HBeAg- chronic hepatitis B	+
cHBV	m	24	Entecavir (since 2016)	39	32	31	+	HBeAg+/(NUC-related) chronic hepatitis B	+
cHBV	f	0	Entecavir (since 2007)	67	31	20	+	HBeAg+/(NUC-related) chronic hepatitis B	n.a.
cHBV	m	0	Entecavir (since 2007)	59	32	39	-	HBeAg- chronic hepatitis B	+
cHBV	m	21	Tenofovir (since 2010)	39	28	47	n.a.	n.a.	+
cHBV	m	0	Entecavir (since 2013)	28	18	31	-	HBeAg- chronic hepatitis B	+
cHBV	m	0	Entecavir (since 2018)	37	34	32	+	HBeAg+/(NUC-related) chronic hepatitis B	+
cHBV	f	265614098	Entecavir (since 2009)	33	53	123	+	HBeAg+/(NUC-related) chronic hepatitis B	+
cHBV	m	345	Entecavir (since 2019)	25	37	25	-	HBeAg- chronic hepatitis B	+
cHBV	m	0	Viread (since 2014)	61	22	19	-	HBeAg- chronic hepatitis B	+
cHBV	f	2243	naive	37	17	15	+	HBeAg+/(NUC-related) chronic hepatitis B	+
cHBV	f	110	naive	35	18	25	-	HBeAg- chronic hepatitis B	-
				41.4 (Mean)					

Supplementary table 9: cHBV/cHDV patient cohort for TRAIL and CD69 analysis

Diagnose	Gender	HBV-PCR [IU/ml]	Anti-HDAg IgG	HDV-PCR [IU/ml]	Therapy	Age	GOT [U/I]	GPT [U/I]	HBeAg+	clinic phase	CMV status
cHBV/cHDV	m	22486	yes	24607	naive	59	54	63	neg	HBeAg- chronic hepatitis B	n.a.
cHBV/cHDV	f	14052	yes	100000	Nuc	51	63	58	pos	HBeAg+/(NUC-related) chronic hepatitis B	+
cHBV/cHDV	m	<10	yes	457980	naive	46	26	35	neg	HBeAg- chronic hepatitis B	n.a.
cHBV/cHDV	m	<10	yes	457980	naive	41	90	110	n.a.	n.a.	n.a.
						49.3 (Mean)					

2. Supplementary figures

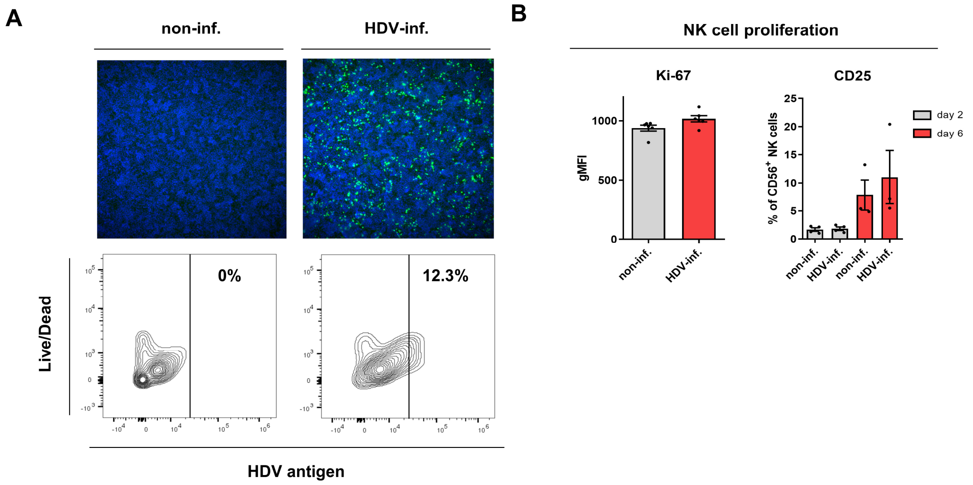

Supplementary Fig. 1. (A) Intracellular expression of the HDV antigen by HepG2-hNTCP cells determined by flow cytometry and immunofluorescence staining (blue: nuclei; green: HDVAg^{+} cells) 7 days post-infection. **(B)** Intracellular expression of Ki-67 (n=6) and surface expression of CD25 (n=3) on healthy donor NK cells in co-culture with non-infected or HDV-infected HepG2-hNTCP cells after 2 days or 2 and 6 days of co-culture, respectively) (bars represent the mean geometric fluorescence intensity or mean frequency of positive cells +/- SEM).

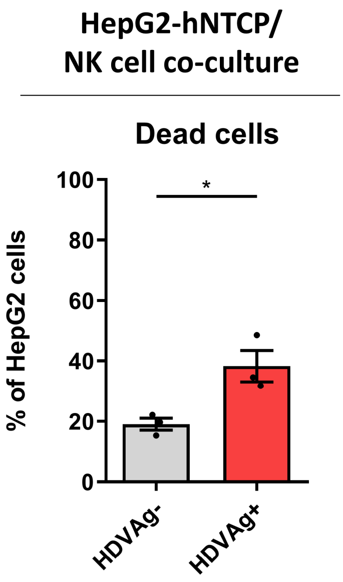

Supplementary Fig. 2. Frequency of dead (fixable viability dye^{+}) HDV-infected HepG2-hNTCP cells in co-culture with NK cells after 48 h (n=3). Data was stratified based on the intracellular expression of HDVAg for analysis (bars represent the mean frequency of positive cells +/- SEM). Levels of significance: *p<0.05 (student's t-test).

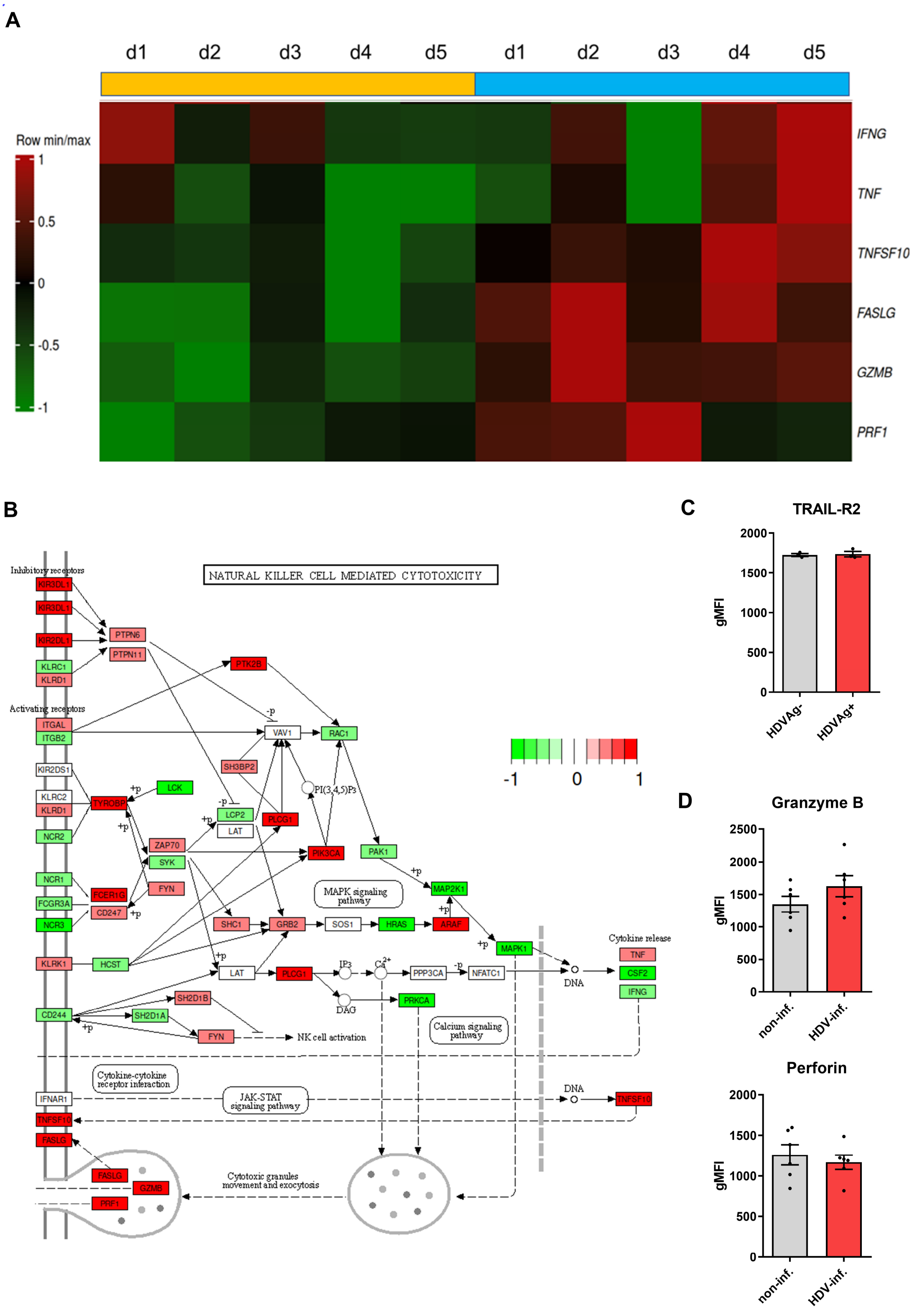

Supplementary Fig. 3. (A) Heatmap showing the expression of the cytotoxicity-associated genes IFN-γ, TNF-α, TNFSF10 (TRAIL), FASLG, GZMB (granzyme B), and PRF1 (perforin) by peripheral NK cells from healthy donors in co-culture with non-infected (yellow) or HDV-infected (blue) HepG2-hNTCP cells (d: donor) (n=5). **(B)** KEGG-based pathway analysis of NK cell cytotoxicity in co-culture with HDV-infected HepG2-hNTCP cells (n=5). Upregulation of genes is displayed in red, down-regulated genes are shown in green. **(C)** Expression of TRAIL-R2 by HDV-infected HepG2-hNTCP cells (n=3) 7 days after infection. Data was stratified based on the intracellular expression of HDVAg for analysis (bars represent the mean geometric fluorescence intensity of positive cells +/- SEM). **(D)** Intracellular expression of perforin and granzyme B (n=6) after 2 days of co-culture with non-infected or HDV-infected HepG2-hNTCP cells (bars represent the geometric mean of the fluorescence intensity of positive cells +/- SEM).

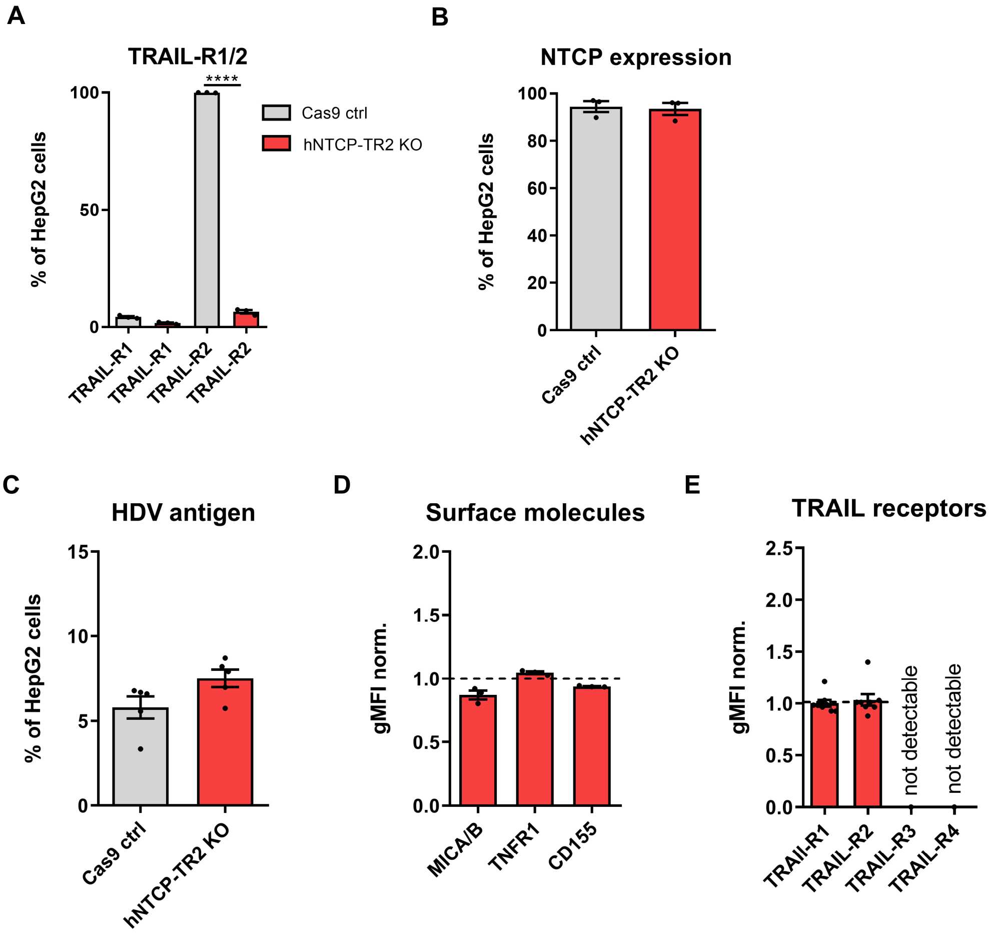

Supplementary Fig. 4. (A) Surface expression of TRAIL-R1 and 2 by HepG2-hNTCP Cas9 ctrl or TRAIL-R2 KO HepG2-hNTCP cells (hNTCP-TR2 KO) (n=3) (bars represent the mean frequency of positive cells +/- SEM). **(B)** Surface expression of hNTCP by HDV-infected Cas9 ctrl or TRAIL-R2 KO HepG2-hNTCP cells (hNTCP-TR2 KO) (n=3) (bars represent the mean frequency of positive cells +/- SEM). **(C)** Intracellular expression of the HDV antigen by HDV-infected Cas9 ctrl or TRAIL-R2 KO HepG2-hNTCP cells (hNTCP-TR2 KO) (n=5) (bars represent the mean frequency of positive cells +/- SEM). **(D)** Surface expression of MICA/B, TNFR1 and CD155 by TRAIL-R2 KO HepG2-hNTCP cells (n=3) (bars represent the geometric mean fluorescence intensity normalized to the mean of HepG2-hNTCP Cas9 ctrl cells +/- SEM). **(E)** Surface expression of TRAIL-R1;2;3;4 on HDV-infected HepG2-hNTCP cells (n=8) (bars represent the geometric mean fluorescence intensity normalized to the mean of non-infected cells +/- SEM). Levels of significance: ****$p<0.0001$ (student's t-test).

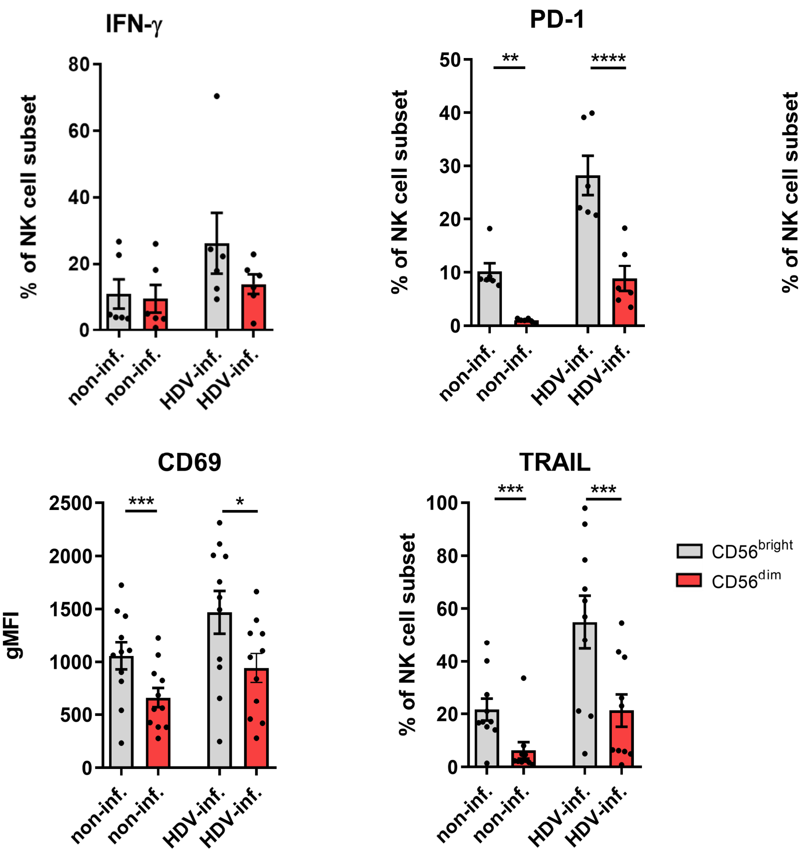

Supplementary Fig. 5. Intracellular expression of IFN-γ (n=6) and surface expression of PD-1 (n=6), CD107a (n=9), CD69 (n=11) or TRAIL (n=10) on NK cells after 2 days of co-culture with non-infected or HDV-infected HepG2-hNTCP cells. NK cells are stratified based on the expression of CD56 on NK cells into $CD56^{bright}$ and $CD56^{dim}$ NK cells for analysis (n=10) (bars represent the geometric mean fluorescence intensity or mean frequency of positive cells +/- SEM). Levels of significance: *p<0.05, **p<0.01, ***p<0.001, ****p<0.0001 (student's t-test).

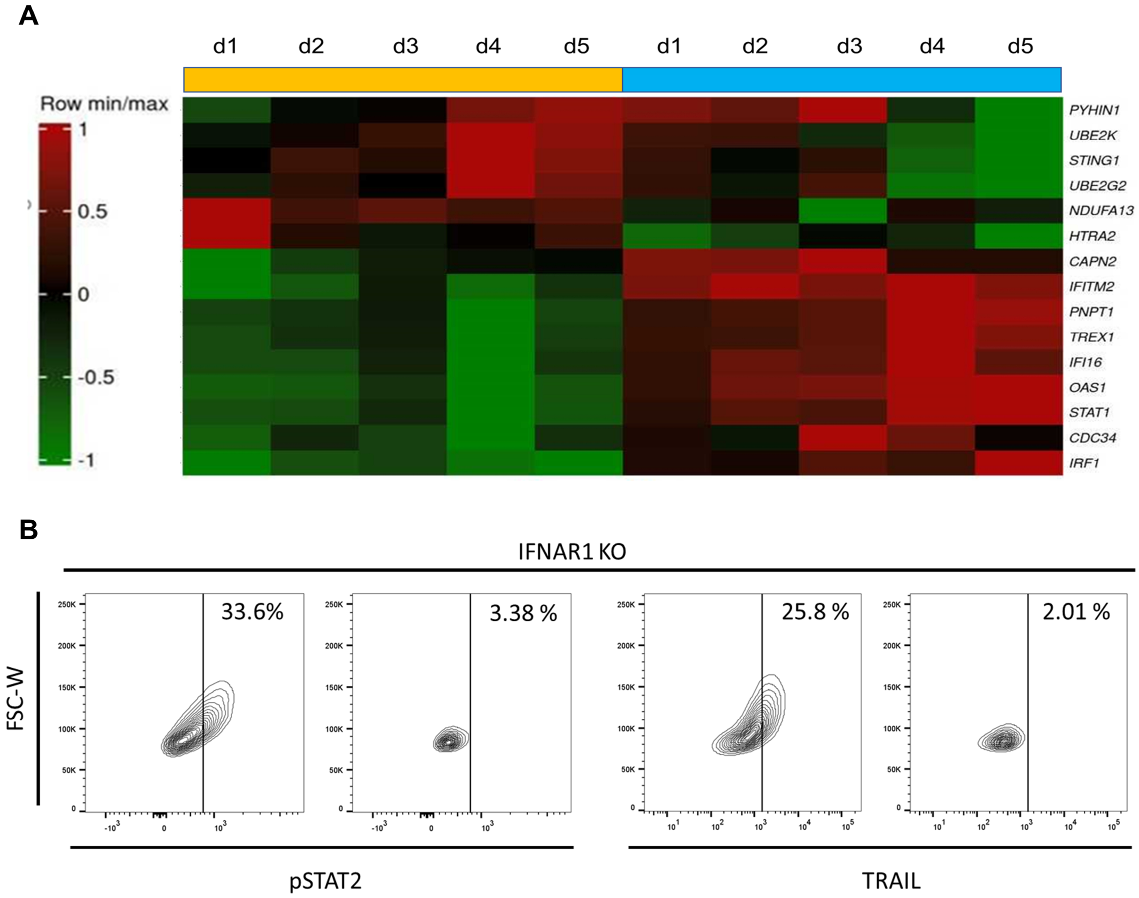

Supplementary Fig. 6. (A) Heatmap showing the expression of IFN-β-associated genes by peripheral healthy donor NK cells in co-culture with non-infected (yellow) or HDV-infected (blue) HepG2-hNTCP cells (d: donor) (n=5). **(B)** Contour plot for the expression of pSTAT2 and TRAIL by transfected (Atto-550^{+}) Mock or IFNAR1 KO NK cells after 24 h of stimulation with IFN-β (100 ng/ml).
